# Field studies on breeding sites of *Culicoides*Latreille (Diptera: Ceratopogonidae) in agriculturally used and natural habitats

**DOI:** 10.1038/s41598-021-86163-9

**Published:** 2021-05-11

**Authors:** Daniela Kameke, Helge Kampen, Alexander Wacker, Doreen Werner

**Affiliations:** 1 Working Group Biodiversity of Aquatic and Semiaquatic Landscape Features, Leibniz Centre for Agricultural Landscape Research (ZALF), Eberswalder Str. 84, 15374 Müncheberg, Germany; 2 Institute of Infectology, Friedrich-Loeffler-Institute (FLI), Südufer 10, 17493 Greifswald, Germany; 3 Zoological Institute and Museum, University of Greifswald, Loitzer Str. 26, 17489 Greifswald, Germany

**Keywords:** Ecology, Entomology, Biodiversity

## Abstract

*Culicoides* are vectors of pathogens mainly of veterinary importance. To establish targeted vector control measures, it is paramount to comprehend the ecological factors determining their distribution. Therefore, we used emergence traps to sample eight biotopes and assess their potential as breeding sites. Part one of the study investigates agricultural habitats, part two compares four biotopes of a forest-dominated area with less anthropogenic influence, including a physicochemical analysis of soil moisture, pH value and organic content. Thirteen culicoid species were collected, with a strong dominance of the Obsoletus Complex on meadows, and with *Culicoides punctatus* (Meigen), *Culicoides pictipennis* (Staeger) and the Obsoletus Complex, to be the most abundant species in the natural habitats. Several co-existing species were found, some of them not having been described before. Our results suggest that ungrazed meadows seem unsuitable as breeding sites. Only the influence of livestock creates adequate conditions for certain midge species. The alder on fen site contained most culicoid species with the highest species diversity. Our study clearly indicates that knowledge of species-specific preferences for environmental habitat conditions (choice of breeding site) in connection to soil conditions is crucial to understand the biology and phenology of midges and their role as vectors of pathogens.

## Introduction

*Culicoides* (Diptera: Ceratopogonidae) are known vectors of arthropod-borne viruses like African horse sickness virus (AHSV), bluetongue virus (BTV) (both genus *Orbivirus*, family Reoviridae) or Schmallenberg virus (SBV) (genus *Orthobunyavirus*, family Peribunyaviridae)^1,2^. After the outbreak of bluetongue disease in 2006 and Schmallenberg disease in 2011 in Central and northern Europe, it became apparent how little was known about the biology and ecology of the viral vectors especially with respect to *Culicoides* breeding sites and their physicochemical characteristics^3,4^. However, to be able to establish targeted vector control strategies, it is crucial to understand the biology and phenology of the species, their choice of breeding sites and the conditions potential breeding substrates must provide.

The genus *Culicoides* Latreille consists of about 96% of haematophagous species obligatorily feeding on mammals^1^.

In contrast to other dipteran families like Culicidae or Simuliidae (both order Diptera), Ceratopogonidae do not contain just aquatic breeders, but also semi-aquatic and terrestrial forms. Most species of the genus *Culicoides* are semi-aquatic as larvae and breed in a wide range of habitats, showing one common feature—a relatively high level of water content^5,6^. As most culicoid species appear to be confined to one type of habitat^5^, biotic and abiotic factors other than soil moisture might have an impact on the choice of breeding sites, too. Culicoid larvae feed on organic matter like fungi, algae or rotten plants^7^, or are predatory on rotifers, nematodes and larvae of other invertebrates^8,9^. Therefore, the organic content of the soil and its compounds have been addressed in various studies as putative co-determining abiotic factors^3,10^. Another parameter considered as potentially influential regarding the quality of breeding substrates is the pH value of the soil^11^. Nevertheless, the overall knowledge about the specific requirements on the breeding substrate of many culicoid midge species is still very scarce.

In the present study, we examine the breeding preferences of *Culicoides* spp. by investigating eight biotopes and conducting a physicochemical analysis of four breeding substrates. The investigation consists of two parts to compare biotopes of high anthropogenic influence with natural habitats. Study one addresses the influence of cattle and sheep on the quality of meadows as potential breeding sites (agricultural habitats). Study two focusses on the suitability of four biotopes in a forest-dominated area and includes a physicochemical soil analysis to define the species-specific breeding conditions. The phenology of the sampled culicoid species is presented.

## Results

### Samples and species composition

Altogether, 547 usable samples were collected and sorted for *Culicoides*. Due to the loss or poor quality of samples, several ones had to be excluded from the study. Reasons include heavy winds or intense UV-light damaging the emergence traps, wild animals dropping the collection boxes, and people damaging or stealing traps/parts of traps.

**Figure 1:**
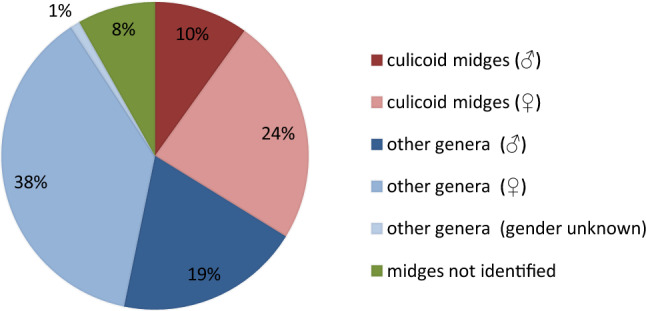
Composition of ceratopogonids sampled in all study sites.

137 samples were collected from regions 1 and 2, while region 3 was represented by 410 samples (Table 1). The total number of midges accounted for 293 individuals. While 170 specimens (110 females, 57 males; gender not determinable in 3 specimens) were species of genera other than *Culicoides* (Fig. 1), 99 belonged to 13 culicoid species (Table 2). Twenty-four midges could only be classified as Ceratopogonidae; bad condition prevented closer determination, even to genus level.

**Table 1:** Details of sampling activities during 2014 including sampling periods, sampling sites with corresponding number of valid samples and substrate analysis.

Biotopes	Sampling period	No. of samples	Coordinates	Soil analysis
**Region 1**				
Ungrazed meadow	April–early Aug	44	N 52.761	
			E 14.306	
Meadow with cattle	April–late July	30	N 52.763	
			E 14.300	
**Region 2**				
Meadow with cattle	Aug–Oct	28	N 52.543	
			E 14.201	
Meadow with sheep	Aug–Oct	35	N 52.502	
			E 14.129	
**Region 3**				
Coniferous woodland	April–Oct	109	N 52.991	Moisture, pH, organic content
			E 12.908	
Deciduous woodland	April–Oct	103	N 52.991	Moisture, pH, organic content
			E 12.90	
Alder on fen site	April–Oct	100	N 52.991	Moisture, pH, organic content
			E 12.907	
Marsh area (grassland)	April–Oct	98	N 52.992	Moisture, pH, organic content
			E 12.903

**Table 2:** Total numbers of *Culicoides* spp. and gender composition from all study sites.

Species	Males	Females	Total
Obsoletus complex	12	33	45
incl. *C. chiopterus* (Meigen), 1830	8	0	8
incl. *C. obsoletus* s.s. (Meigen), 1818	2	3	5
*C. achrayi Kettle* and Lawson, 1955	0	1	1
*C. albicans* (Winnertz), 1852	1	7	8
*C. comosioculatus* Tokunaga, 1956	0	1	1
*C. grisescens* Edwards, 1939	0	7	7
*C. impunctatus* Goetghebuer, 1920	2	5	7
*C. kibunensis* Tokunaga, 1937	0	3	3
*C. pallidicornis* Kieffer, 1919	0	2	2
*C. pictipennis* (Staeger), 1839	5	4	9
*C. pulicaris* (Linnaeus), 1758	4	0	4
*C. punctatus* (Meigen), 1804	5	5	10
*C. subfagineus* Delécolle & Ortega, 1998	0	1	1
*C. subfasciipennis* Kieffer, 1919	0	1	1
Total	29	70	99

Of the 13 identified species, the Obsoletus Complex revealed the highest number with 45 specimens. Morphological identification of intact male specimens and the molecular analysis of females determined 13 of the Obsoletus Complex specimens as *C. obsoletus* s.s. and *C. chiopterus*.

The maximum number of individuals collected from the same species (*Culicoides punctatus* (Meigen), 1804) was 10 (Table 2). Most *Culicoides* (70.7%) were females, with a 2.4:1 ratio of female:male).

Four Obsoletus Complex females were gravid. These comprise specimens sampled on coniferous woodland (CW) in June (1 specimen), meadow with cattle in June (2 specimens) and meadow with sheep in August (1 specimen).

#### Study 1: influence of cattle and sheep on meadows

Thirty-three individuals were collected on all four meadows with the highest number of *Culicoides* per sample being found on the meadow with cattle in region 1. The ungrazed meadow revealed no *Culicoides*.

Later in the year, many fewer *Culicoides* per sample were collected on the meadow with cattle in region 2. The mean number of *Culicoides* collected on the meadow with sheep was even smaller (Table 3).

**Table 3:** Quantitative composition of culicoid biting midges per biotope.

Biotope	No. of samples	No. of specimens	No. of specimens per sample	No. of species
**Region 1**				
Ungrazed meadow	44	0	0.00	0
Meadow with cattle	30	25	0.83	2
**Region 2**				
Meadow with cattle	28	6	0.21	1
Meadow with sheep	35	2	0.06	1
**Region 3**				
Coniferous woodland	109	8	0.07	4
Alder on fen site	100	40	0.40	10
Deciduous woodland	103	12	0.12	4
Marsh area	98	6	0.06	4
Total/mean	Σ 547	Σ 99	Ø 0.22	Ø 3.3

Mainly specimens of the Obsoletus Complex were sampled on meadows. Additionally, one individual of *C. comosioculatus* Tokunaga 1956 was collected on the meadow with cattle in region 1 (Table 4).

**Table 4:** Number of collected culicoid species per biotope and sample.

	Marsh area	Alder on fen site	Deciduous woodland	Coniferous woodland	Meadow (ungrazed)	Meadow (cattle)	Meadow (sheep)
*C. achrayi*	0.00	0.01	0.00	0.00	0.00	0.00	0.00
*C. albicans*	0.00	0.06	0.02	0.00	0.00	0.00	0.00
*C. comosioculatus*	0.00	0.00	0.00	0.00	0.00	0.02	0.00
*C. grisescens*	0.00	0.03	0.01	0.03	0.00	0.00	0.00
*C. impunctatus*	0.00	0.05	0.00	0.02	0.00	0.00	0.00
*C. kibunensis*	0.01	0.02	0.00	0.00	0.00	0.00	0.00
Obsoletus Complex	0.02	0.08	0.01	0.02	0.00	0.52	0.06
*C. pallidicornis*	0.02	0.00	0.00	0.00	0.00	0.00	0.00
*C. pictipennis*	0.00	0.01	0.08	0.00	0.00	0.00	0.00
*C. pulicaris*	0.00	0.04	0.00	0.00	0.00	0.00	0.00
*C. punctatus*	0.00	0.09	0.00	0.01	0.00	0.00	0.00
*C. subfagineus*	0.01	0.00	0.00	0.00	0.00	0.00	0.00
*C. subfasciipennis*	0.00	0.01	0.00	0.00	0.00	0.00	0.00
midges per sample and biotope	0.06	0.40	0.12	0.07	0.00	0.53	0.06

#### Study 2: biotopes in a forest-dominated area

In total, 66 *Culicoides* were caught within the four biotopes of region 3. The alder on fen site (AFS) yielded the highest species diversity (10 species), the highest total number of *Culicoides* and the highest number of individuals per sample of region 3 (Table 3). It also presented the highest number of specimens of a single taxon (*C. punctatus*, closely followed by the Obsoletus Complex). The remaining three biotopes contained four species each in different compositions, of which only *C. pictipennis* (Staeger) 1839 on the deciduous woodland (DW) reached a relatively high value per sample (Table 4).

### Soil analysis in forest-dominated biotopes

**Figure 2:**
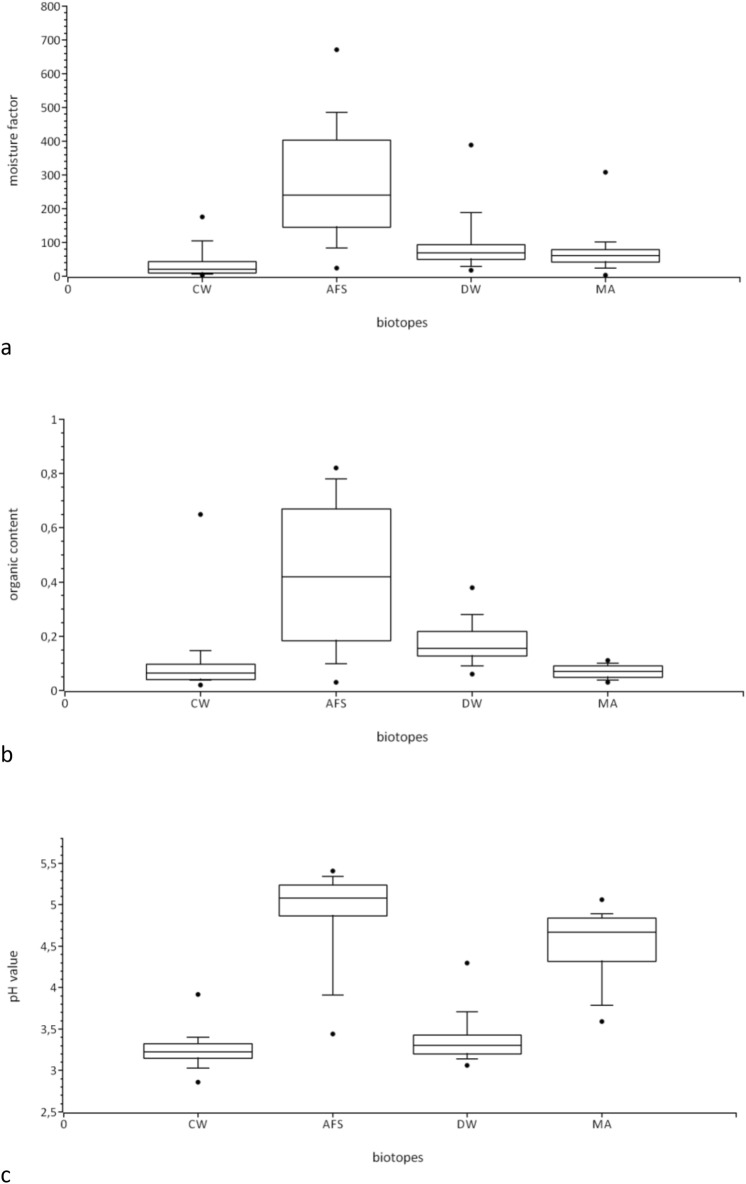
All box plots (**a**–**c**) comprise 25th and 75th percentiles (whisker box) and include the median (central line). Error bars represent 10th and 90th percentiles, with the dots delineating minimum and maximum data points. (**a**) Boxplots of the soil moisture factors measured in the four biotopes (*CW* Coniferous woodland, *AFS* Alder on fen site, *DW* Deciduous woodland, *MA* Marsh area) in region 3. (**b**) Boxplots of the organic contents measured in the four biotopes (*CW* Coniferous woodland, *AFS* Alder on fen site, *DW* Deciduous woodland, *MA* Marsh area) in region 3 (values between 0–1 correspond to 0–100%). (**c**) Boxplots of the pH values measured in the four biotopes. (*CW* Coniferous woodland, *AFS* Alder on fen site, *DW* Deciduous woodland, *MA* Marsh area) in region 3.

The distribution of the measured soil factors in each biotope of region 3 are illustrated in Fig. 2a–c.

The AFS displayed the highest maximum values, but also the widest variances regarding all soil factors. It also contained the highest inter-quartile ranges for soil moisture and organic content, as does the marsh area (MA) for pH value.

Especially accounting for soil moisture, less for organic content, the inter-quartile ranges of the CW, DW and MA were on an almost equally low level. Merely the pH values of the MA showed intermediate character compared to the AFS on the one side and the CW and DW on the other side.

### Statistical analysis

None of the three soil factors nor the number of collected *Culicoides* spp. was normally distributed.

The Kruskal–Wallis-test revealed that the biotopes were significantly different from each other regarding the three soil factors (with χ^2^ = 29.86, df = 3, *p* < 0.001 for moisture; χ^2^ = 44.24, df = 3, *p* < 0.001 for pH, and χ^2^ = 46.12, df = 3, *p* < 0.001 for organic content). The AFS showed the highest means of each analysed soil factor (Table 5).

**Table 5:** Means of each soil factor per biotope.

	Coniferous woodland	Alder on fen site	Deciduous woodland	Marsh area
Soil moisture	36.74	274.79	92.24	65.19
pH	3.27	4.79	3.38	4.53
Organic content	9	36	16	7

The number of *Culicoides* differed significantly between the four biotopes of region 3, with χ^2^ = 17.419, df = 3, and *p* = 0.001, as revealed by the Kruskal–Wallis-test. The median test clearly illustrates that most biting midges were captured within the AFS during most sampling days (Table 6).

**Table 6:** Results of the median test on the number of collected *Culicoides* per biotope.

	Coniferous woodland	Alder on fen site	Deciduous woodland	Marsh area
**No. of biting midges**				
> Median	3	12	4	3
≤ Median	10	1	9	10

Logistic regression analysis showed that each soil factor had an influence on the probability of *Culicoides* to be present or not in region 3 (Omnibus test χ^2^ = 25.95, df = 1, *p* < 0.001 for moisture; χ^2^ = 8.88, df = 1, *p* < 0.001 for pH, and χ^2^ = 14.59, df = 1, *p* < 0.001 for organic content).

While the soil characteristics were important for presence and absence, we found no significant interrelations between the three soil factors and the number of collected *Culicoides* (linear regression model): As the bivariate correlation analysis after Pearson revealed strong correlations between the three soil factors (moisture-pH: *r* = 0.686, *p* < 0.001; moisture-organic content: *r* = 0.915, *p* < 0.001; pH-organic content: *r* = 0.502, *p* < 0.001) and weaker correlations between any soil factor and the number of sampled *Culicoides* (*Culicoides*-moisture: *r* = 0.385, *p* = 0.014; *Culicoides*-pH: *r* = 0.242, *p* = 0.084; *Culicoides*-organic content: *r* = 0.274, *p* = 0.050), an interpretation of regression coefficients is not reasonable.

### Biodiversity indices

Table 7 shows the calculated diversity indices and reveals huge differences regarding the evaluated biodiversity between the three agriculturally used habitats of regions 1 and 2 and the four more natural biotopes (AFS, MA, CW, DW).

**Table 7:** Biodiversity indices of Shannon–Weaver, Evenness and Simpson index.

	Region 1	Region 2		Region 3			
	Meadow with cattle	Meadow with cattle	Meadow with sheep	Coniferous woodland	Deciduous woodland	Alder on fen site	Marsh area
No. of specimens (n)	25	6	2	8	12	40	6
No. of species (S)	2	1	1	4	4	10	4
Shannon–Weaver index (H)	0.24	0	0	1.91	1.42	2.96	1.92
Maximum diversity possible (H_max_)	1.00	0	0	2.00	2.00	3.32	2.00
Evenness (E)	0.24	–	–	0.95	0.71	0.89	0.96
Simpson index (D)	0.92	1.00	1.00	0.18	0.44	0.13	0.13

Index numbers and calculated biodiversity indices of Shannon–Weaver index and Simpson index based on *Culicoides* spp. collected in 2014.

The meadows of region 2 contained only one species each, resulting in the minimum Shannon–Weaver index of zero (no diversity). The meadow with cattle of region 1 has also a very low Shannon–Weaver index (0.24) and, with two species present, an Evenness of 0.24. The Simpson index of all three meadows is either D = 1 or little less, also expressing the lack of biodiversity within these biotopes (Table 7).

Compared to the agriculturally used habitats, the four biotopes of region 3 contained more species, but with varying numbers of collected specimens. The Shannon–Weaver indices range between 1.42 (DW) and 2.96 (AFS). Population numbers of sampled *Culicoides* species were most equally balanced in the MA (0.96) and CW (0.95), closely followed by the AFS (0.89). The DW revealed a lower Evenness factor of 0.71, disclosing the dominance of one or few species in this biotope. While the Simpson index depicts the AFS and MA as the two biotopes containing the highest biodiversity of region 3 with D = 0.13, the DW reveals much less diversity reaching only D = 0.44 (Table 7).

### Seasonal distribution/phenology

**Figure 3:**
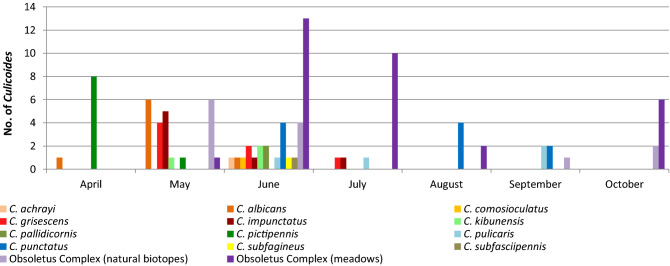
Phenology of *Culicoides* spp. based on all study sites sampled during 2014.

*Culicoides* emerged throughout the sampling period between April and October 2014. The months May and June produced most midges and the highest species diversity. The Obsoletus Complex was present for six months from May until October. Other species appeared only for a short period of time (Fig. 3).

We observed several co-habitating culicoid species, all sharing the same biotope as a developmental site (Table 8). For example, *C. albicans* occurred together with *C. pictipennis* in the DW. However, as expected, the AFS revealed most of the co-existing species with a peak in June.

**Table 8:** Co-occurrence and species distribution per month and biotope of *Culicoides* spp. collected during 2014.

Time of appearance	Coniferous woodland	Deciduous woodland	Alder on fen site	Marsh area
Mid-April			*C. pictipennis*	
Late April		*C. albicans*^a^ *C. pictipennis*^a^		
Mid-May		*C. albicans* *C. pictipennis*	Obsoletus Complex *C. albicans* *C. obsoletus* s.s	
Late May	*C. grisescens* *C. impunctatus*	Obsoletus Complex	Obsoletus Complex *C. albicans* *C. grisescens* *C. impunctatus* *C. kibunensis*	Obsoletus Complex
Mid-June	Obsoletus Complex *C. grisescens* *C. punctatus*	*C. grisescens*	*C. albicans*^a^ *C. kibunensis*^a^	Obsoletus Complex
			Obsoletus Complex^a^ *C. impunctatus*^a^	
			*C. punctatus*^a^ *C. subfasciipennis*^a^	
Late June			*C. achrayi*^a^ *C. pulicaris*^a^	*C. kibunensis* *C. pallidicornis* *C. subfagineus*
Mid-July	*C. impunctatus*		*C. grisescens*	
Late July			*C. pulicaris*	
Mid-Aug			*C. punctatus*	
Late Aug			*C. punctatus*	
Mid-Sep			*C. pulicaris*^a^ *C. punctatus*^a^ *C. obsoletus* s.s	
Late Sep			*C. pulicaris* *C. punctatus*	
Mid-Oct			*C. obsoletus* s.s	

^a^Species collected in one single sample.

## Discussion

In total, 13 culicoid species were found in the present study, with 45.5% of the collected specimens belonging to the Obsoletus Complex while species only occasionally present in previous collections in Germany, accounted for approximately 25% of the sampled individuals. Thus, the species composition is only partly in accordance to earlier studies on the German *Culicoides* fauna according to which 70 to over 90% of the specimens belonged to the Obsoletus Complex and up to 20% represented members of the Pulicaris Complex, while other culicoid species were present in negligible numbers only^12,13^. However, previous studies were based on UV-light trap catches^12–15^ and targeted active culicoid specimens^16^. The results obtained in this study are very specific as they represent the species compositions associated with the respective breeding substrates.

The gender ratio differed strongly between species, revealing no pattern applicable to all species. The dominance of female *Culicoides* emerging from breeding sites corresponds to earlier results^17,18^, even though the sex ratio in the present study showed a much higher proportion of females with 70.7% or a female:male ratio of 2.4:1 than the above studies with 55.6%^17^ or a female:male ratio of 1.06:1^18^.

The evaluation of the diversity of each biotope (excluding the ungrazed meadow where no *Culicoides* were found) revealed clear differences between the agriculturally used habitats and the more natural biotopes. The Shannon–Weaver index depicted very low diversity for all three studied meadows where biting midges were found. The two meadows (with cattle and sheep) of region 2 reached the lowest possible diversity. This seems plausible as only one species was sampled within each biotope. The meadow with cattle of region 1 revealed at least two species. The Evenness factor of 0.24 depicts the dominance of one of them. The low number of species and unbalanced number of specimens within the biotope result in a low Shannon–Weaver index of 0.24, which describes the poor level of biodiversity.

The Simpson index measures the probability that two individuals, randomly selected from a sample, belong to the same species. As only one species was sampled on each meadow from region 2, the probability to choose two specimens which belong to one species is 100% (displayed by the value of D = 1.0). The meadow with cattle of region 2 revealed at least two culicoid species, but the dominance of one species leads to a high Simpson index of 0.92 as well.

Opposite to the very low biodiversity of all meadows, the four more natural biotopes of region 3 show an overall high level of biodiversity: according to the Shannon–Weaver index, the level of biodiversity is highest within the AFS (H = 2.96). Compared to the other biotopes of region 3, the AFS revealed by far the highest numbers of culicoid species and specimens. This and the relatively high Evenness factor (E = 0.89) lead to the high H value. The Shannon–Weaver indices for CW and MA are 1.91 and 1.92, respectively. Based on the low numbers of species and specimens in both biotopes, the relatively high H value is mainly caused by its high Evenness values of 0.95 (CW) and 0.96 (MA), respectively. Therefore, the almost equal numbers of all present species leads to the relatively high biodiversity, rather than a high number of species.

The Shannon–Weaver index of the DW is the lowest of the four biotopes of region 3 with H = 1.42 and rates this biotope as the one with the lowest diversity of region 3. Though the number of species equal the one of the CW and MA, the higher number of specimens and especially the much lower Evenness factor of 0.71 reduces the H value.

Other than the Shannon–Weaver index, the Simpson index rates both, the AFS and the MA, as the two most diverse biotopes. With values of D = 0.13, the probability to randomly select two species of the same species is rather low in both biotopes. As the AFS revealed more than double as many species than the MA, the lower number of caught specimens of the MA must have led to the same biodiversity rate.

*Study 1—Influence of domestic animals on meadows*: up to date, dung-breeding *Culicoides* have been investigated more thoroughly^18–20^ than most other culicoid species. Most studies have focused on examining selectively either dungheaps or cowpats, rather than conducting a direct comparison between grazed and ungrazed meadows under field conditions. In the present study, we were able to show that the ungrazed meadow seems to be an unsuitable breeding habitat for *Culicoides*. Therefore, it seems plausible that the suitability of meadows as culicoid breeding sites can be largely, if not completely, attributed to the influence of livestock pasturing.

The strong dominance of Obsoletus Complex specimens sampled on grazed meadows is not surprising as this species complex is known to contain typical dung-breeders^19,20^. The high potential of manure as a breeding substrate has been demonstrated before^21,22^ and explains the high quantity of *Culicoides* developing on meadows used by cattle in the present study. While 0.83 midges/sample were found on the meadow with cattle in region 1, only 0.21 midges/sample were collected on the meadow with cattle in region 2. The quantitative differences between these two study sites might be caused by the differing time periods of sampling (April to July for region 1 and August to October for region 2). Previous studies observed population peaks of Obsoletus Complex midges in October, though^23^, giving reason to expect even higher numbers of midges for region 2 than for region 1, particularly so, as region 2 is an agriculturally dominated area with a higher abundance of potential blood hosts and more suitable breeding habitats than region 1.

Compared to the much higher total number of midges emerging from cowpats, sheep dung produced only two specimens. The very low number of midges originating from sheep faeces might be due to the very quick decomposition and desiccation of the rather small droppings, which likely reduces the quality of these remains as culicoid breeding sites. Therefore, it can be assumed that, contrary to pastures with cattle dung, sheep-runs might not play an essential role in promoting the distribution of *Culicoides*. For modeling approaches, it should be considered, though, that this might only apply to single scattered pieces of faeces as the longer persistence of higher volumes of sheep dung, i.e. on muckheaps, might very likely raise its quality as potential breeding sites as observed by^21^.

All grazed meadows revealed very few culicoid species. Besides members of the Obsoletus Complex, only one individual of *C. comosioculatus* was found. The present investigation represents a case study though as merely one habitat of each type was sampled. More research to confirm the present results is therefore strongly recommended, even more, as ceratopogonid communities of terrestrial ecosystems have been barely investigated^24^, with the consequence that breeding sites of *Culicoides* spp. are still poorly known^25^.

*Study 2—Quality of forest-dominated biotopes as culicoid breeding sites*: In the present study, the AFS turned out to be very productive as a culicoid breeding site in regards to the number of caught specimens and species diversity. Ten of the 13 collected species were found in the AFS. This is 2.5 times as many species as in the three other biotopes of region 3, which contained four species each in different compositions. Therefore, species-specific requirements for larval development seem to be met for more culicoid species in the AFS than in any of the other study sites.

The measured pH values are in accordance to soil analyses conducted in German forests^26^. As the top layers usually are the most acidic ones, the chosen depth of soil sampling in the present study (upper 0–5 cm) persistently produced low pH values. Additionally, the used solvent (CaCl_2_) is less sensitive to fast changing weather conditions, but also lowers the measured pH value significantly compared to distilled water^26^—a solvent often used in earlier studies analyzing the distribution of Ceratopogonidae.

The wide variances of the soil factors, especially moisture and organic content, were mainly caused by unequal soil conditions within each biotope rather than changes over time (unpublished data). Nevertheless, the statistical analysis revealed that all four biotopes of region 3 were significantly different from each other regarding the three soil factors. Comparing the means of each soil factor revealed that the AFS contained a higher level of soil moisture, a less acidic pH value and a higher organic content than the other three biotopes of region 3. We could show that significantly more midges (0.4 *Culicoides*/sample) developed in the AFS compared to the three other biotopes of region 3 with 0.12 (DW), 0.07 (CW) and 0.06 (MA) *Culicoides* per sample.

Previous studies have assumed that the level of moisture be a crucial factor for ceratopogonid development^17,20^. Also, some studies determined the organic content as pivotal^17,27^. Our statistical analysis revealed that each soil factor has an impact on the probability of *Culicoides* to occur. Due to high correlations between the various measured soil factors, it could not be clarified, though, whether they influence the number of specimens, too. But as many culicoid species are known to lay their eggs in batches and previous egg-laying encourages females to oviposit at the same site^28^, an increase in the probability of biting midge presence should indirectly result in a higher number of specimens, too.

The aggregation of larvae in terrestrial habitats^29^ typically results in a high number of samples completely devoid of midges and an overall low number of specimens sampled by emergence traps^30^. Thus, the obtained low numbers of collected specimens are not surprising. Nevertheless, emergence traps are still considered to be the best tool for the investigation of breeding site productivity, as it offers a safe assignment of species to their specific developmental sites^24,29,31^.

The *Culicoides* collected in this study are discussed on species level in regards to existing literature.

*Culicoides achrayi* was found in the AFS. A swamp as a breeding site^32^ and soil located in stagnant water^22^ have previously been described for this species. We confirm June as the time of emergence^32^ and add that *C. achrayi* co-exists with *C. pulicaris*.

*Culicoides albicans* was collected in the AFS and DW. Specimens hatched from late April to mid-June, representing one generation per year. We confirm co-habitation with *C. pictipennis* and *C. kibunensis*^11,33^ and the preference for very humid substrates which has been described for the wettest parts of boglands^5,34^ and for artificially waterlogged soil^11^. Our results show, that *C. albicans* larvae can tolerate medium moisture levels, too. The mean organic content of their developmental sites reached from moderate to high, and the pH values lay between strong and ultra-acidic.

*Culicoides comosioculatus* was found on the meadow with cattle dung in mid-June. As only one individual (a gravid female with the presumed intention to oviposit) was collected and no literature regarding breeding sites of this species could be found, our finding only indicates that this species might possibly develop in animal dung although in extremely low numbers.

*Culicoides grisescens* was found within the AFS, the CW and the DW from late May until mid-July. Kremer^35^ listed soils of swamps and boggy grasslands as developmental sites. We collected *C. grisescens* in three different biotopes with wide variances of the mean moisture level, mean organic content and mean pH value, which reveals the wide tolerance range of this species towards these three soil factors.

*Culicoides impunctatus* was collected in the AFS and the CW from late May to mid-July, representing one generation per year. This finding differs from earlier observations of two generations per year in Scotland^36^. Previous studies described breeding sites as acidic, oligotrophic grasslands, swamps, boglands or marshes, often of a peaty consistence^5,10,33,34,37^ and with soil pH values of 5.0–6.5 (dissolved in distilled water)^37^. This matches the pH values of the AFS in the present study (lower, but dissolved in CaCl_2_), but excludes the much lower pH values of the CW. The range considered suitable for *C. impunctatus* larvae should therefore be extended downwards to as low as pH 2.9–3.9 (CaCl_2_). We found *C. impunctatus* in two biotopes comprising a wide variance regarding soil moisture and organic content, which illustrates the wide tolerance range of this species. Individuals of *C. impunctatus* co-exist with Obsoletus Complex specimens as both were collected within the same sample in the AFS.

*Culicoides kibunensis* was collected in the AFS and MA, which matches earlier observations depicting swamps of eutrophic fresh water bodies^17,34^, soil of stagnant water bodies^22^ and acidic grasslands in considerable distances to swamps^33^ as breeding sites. The AFS and MA revealed pH values between 3.4 and 5.4. Soil moisture and organic content displayed wide variances. All specimens hatched from late May to mid-June. *Culicoides kibunensis* was found to co-exist with *C. albicans* as observed by Kettle^33^. Earlier observations of co-habitations with *C. obsoletus* s.s. and *C. pallidicornis*^5,34^ could not be confirmed.

*Obsoletus Complex* members were present in all study sites except for the ungrazed meadow. In the grazed meadows, Obsoletus Complex midges emerged almost throughout the entire sampling period except for the month of September. Two peaks were observed, one in June/July and a smaller one in October. As in the grazed meadows, the biotopes of region 3 also revealed two generations, but emerging at a slightly earlier time of the year with one peak in May/June and the other one in September/October.

Members of the Obsoletus Complex are known to be generalists regarding their choice of breeding sites. Only the identified member species, *C. chiopterus* and *C. obsoletus* s.s., are considered here.

*Culicoides chiopterus* was exclusively found on meadows grazed by cattle, which is in accordance to several earlier studies as this species is described as a dung-breeding species developing in cowpats and horse droppings^5,34,35,38^.

*Culicoides obsoletus* s.s. was mostly sampled in the AFS. Only one individual was collected on a meadow grazed by cattle. Previous descriptions of breeding sites differed widely. Acidic grasslands in considerable distance to bogs/swamps^33^ and leaf litter compost^5,35^ could not be confirmed in the present study, although the MA and AFS were of a comparable character. While Uslu and Dik^17^ could not find any *C. obsoletus* s.s. in wet organic matter-rich soil, we collected most specimens of this species in the AFS and can therefore confirm previous findings^11,29,32,39^. The time of *C. obsoletus* s.s. activity in Germany (April–October) as described by Havelka^32^ agrees with our observations.

*Culicoides pallidicornis* was found in the MA in late June. This species revealed the smallest variances of all sampled biting midge species regarding the three soil factors, using soil with pH values of 3.6–5.0 (CaCl_2_) and a relatively low level of moisture. This contradicts earlier observations where *C. pallidicornis* developed in the mud of eutrophic fresh-water swamps^5^. While *C. pallidicornis* larvae are known to co-exist with *C. kibunensis*^5^, we can add *C. subfagineus* to share the same developmental site.

*Culicoides pictipennis* was collected in the DW and, to a minor part, in the AFS. The preferred physicochemical breeding conditions were ultra to extremely acidic with a medium moisture level and a moderate to slightly increased organic content. This differs from previous studies, which have found this species to develop only at the margin of stillwater bodies like pools and ponds, and the littoral of lakes or in artificially waterlogged soil^11,32,34^. Havelka^32^ observed *C. pictipennis* between May and June, while in our investigation the first specimen emerged as early as mid-April. We can confirm the co-existence of *C. pictipennis* and *C. albicans* as previously observed by Harrup^11^.

*Culicoides pulicaris* was sampled in the AFS from late June until September, which agrees with observations denoting May to September as the activity time of this species^32^. *Culicoides pulicaris* seems to prefer breeding substrates with a high moisture level and a high organic content, as previously described^17,32,34^. We can add that *C. pulicaris* breeds in soil showing pH values at least between 4.0 and 5.4. We collected *C. pulicaris* together with *C. achrayi* and found it to simultaneously emerge from one biotope with *C. obsoletus* s.s. Additionally, we can confirm the co-existence of *C. pulicaris* with *C. punctatus*^5,40^, since both species have similar breeding habitat preferences^11^.

*Culicoides punctatus* was sampled in the AFS and, to a minor part, in the CW. Time of emergence was from mid-June to late September, which is in accordance with earlier observations listing April-August and October as times of activity^32^. In the present study, a strong preference for swampy conditions with soil of high moisture, high organic content and a strong to very strong acidity was found. This is in agreement to previous findings^11,32,41^. The co-existence of *C. punctatus* with *C. pulicaris* is well known^5,40^ and can be confirmed once more. Additionally, we found *C. punctatus* to co-occur with *C. subfasciipennis*.

*Culicoides subfagineus* was caught in the MA in late June. The soil was oligotrophic and contained a relatively low moisture level with pH values between 3.6 and 5.0. The first record of this species in Germany was in 2014, when *C. subfagineus* was observed to attack cattle^42^.

*Culicoides subfasciipennis* was sampled in mid-June in the AFS. The time and choice of breeding site are in accordance to previous findings^17,32^. Breeding conditions for the only individual collected revealed a medium soil moisture factor, a pH value of 5.2 and a medium organic content. The species was found to co-develop with *C. punctatus*.

## Conclusion

By conducting a direct on-the-field comparison, we were able to show that ungrazed pastures seem to be unsuitable breeding habitats for biting midges and that solely the use of pastures by domestic animals create appropriate breeding conditions for few culicoid species. While pastures with the influence of cattle produced the highest numbers of specimens, mainly of Obsoletus Complex midges, the influence of sheep was far less productive.

All four “natural” biotopes of study 2, situated in a forest-dominated area, produced less specimens per sample than the pasture with cattle, but a higher species diversity. Most individuals and the highest diversity of culicoid species were found in the AFS, which contained the highest means of organic content, soil moisture and pH value. In each of the biotopes ‘CW’, ‘DW’ and ‘MA’, four biting midge species were collected, but with different species compositions. The number of emerging specimens and species was highest in May and June, with some species being limited to a short time period of appearance (e.g. *C. achrayi*) while other species developed almost throughout the season (e.g. Obsoletus Complex, *C. punctatus*)*.* The 13 collected species differed widely in their choice of breeding sites and therefore also in their breeding substrate preferences. Every single one of the measured soil factors (moisture, organic content, pH value) has a statistical influence on the probability of culicoid midges to occur. To understand the biology and phenology of biting midges and their role as vectors of pathogens, it is of high importance to gain closer knowledge of species-specific preferences for breeding sites in combination with physicochemical properties and the agricultural pasture use.

## Methods

### Insect collection and identification

Insect collection took place during the summer 2014 at several sites in three different regions of the federal state of Brandenburg, Germany (Table 1).

Ten emergence traps (ground area 30 × 30 cm^2^) were positioned randomly within each studied biotope. Collection took place every two weeks, with the traps afterwards being moved to new sites within the biotope. Occasionally the traps captured gravid midges, which must have foraged on the sampled substrate before, or entered the enclosed space during the process of placing the traps, with the intention to oviposit. Therefore, gravid midges were not excluded from the analysis but considered as a representation of the substrate’s potential as a suitable breeding site. Biting midges were collected and stored in ethanol (75%) until further analysis. Identification to species or complex level was conducted under a stereo microscope following the identification keys of Delécolle^43^ and Mathieu et al.^44^. Specimens of poor condition, which could not be identified morphologically, were subjected to molecular analysis by COI barcoding using primers PanCuli-COX1-211F and PanCuli-COX1-727R^45^.

Some of the specimens belonging to the species *Culicoides obsoletus* (Meigen) 1818, *Culicoides scoticus* Downes and Kettle 1952, and *Culicoides chiopterus* (Meigen) 1830, plus the isomorphic species *Culicoides dewulfi* Goetghebuer 1936, which could neither be determined by a morphological nor by molecular approach, are referred to as Obsoletus Complex.

### Study sites

#### Study 1: influence of cattle and sheep on meadows

To determine the influence of cattle and sheep on the quality of meadows as potential breeding habitats of culicoid midges, an ungrazed meadow, meadows with cattle dung and a meadow with sheep dung were investigated in two different regions, for reasons described below. The comparison of an ungrazed meadow with a meadow with cattle dung was conducted in region 1, which was located in the far eastern part of the federal state of Brandenburg, Germany, close to the Oder River on the Polish border and had occasionally been flooded in previous years. The ungrazed meadow had not been used as a pasture for more than ten years. The comparison of the influence of cattle vs. sheep on meadows was conducted in region 2, an agriculturally dominated area, located in the nature reserve Märkische Schweiz, Brandenburg, Germany (Table 1).

Due to the dry summer 2014, the farmer had to use the ungrazed meadow for his livestock animals, which lead to the loss of the ungrazed meadow for this project. A short-termed change of plans was inavoidable and resulted in the second comparison between a meadow with cattle and a meadow with sheep in region 2.

#### Study 2: biotopes of a forest-dominated area

Following the approach described above, insects were sampled in four biotopes of region 3: coniferous woodland (CW), deciduous woodland (DW), alder on fen site (AFS) and marsh area (MA) (Table 1). The area is a forest-dominated territory, positioned in northwestern Brandenburg, Germany, presenting wide patches of deciduous and coniferous woodland. The emergence traps of the AFS were placed along the littoral zone within the muddy area.

### Soil sampling and analysis

In region 3, soil samples were taken to analyse soil parameters such as water content (moisture factor), acidity/alkalinity (pH value) and organic content.

Except for days with heavy rainfall (April and end of June), each soil sample was taken at the beginning of the fortnightly collection period right beside the emergence traps and obtained from the upper layers of substrate (0–5 cm) using a hand shovel. Samples were immediately processed after arrival in the laboratory.

#### Soil moisture factor

Each soil sample was freshly weighed (fresh weight), dried at 105 °C for approximately 24 h to obtain a constant weight and then re-measured (dry weight). The moisture factor was calculated as follows:

$${\text{Moisture}}\;{\text{ factor }} = \, \left( {\left( {{\text{fresh }}\;{\text{weight }} - {\text{ dry }}\;{\text{weight}}} \right) \, /{\text{ dry }}\;{\text{weight}}} \right) \, *{ 1}00$$

#### pH value and organic content

On 12 June, 23 July and 17 September 2014, ten soil samples per biotope were taken as described above. In the laboratory, each sample was immediately dried at 40 °C. After oven-drying, all samples were passed through a 2 mm sieve.

pH-values were measured with a WTW Multimeter 3410 (Weilheim, Germany) using a 0.01 M CaCl_2_ solution (5 ml soil + 25 ml CaCl_2_ solution), following the standard procedure of the HFA 3.1.1.7^46^.

The classification for soil pH values of the United States Department of Agriculture Natural Resources Conservation Service was used^47^.

The remaining sieved soil samples were dried for 24 h at 105 °C to a constant weight and then used for evaluating the organic content following the LOI (loss-on-ignition) method (5 g per sample ashed for 4 h at 550 °C). The organic content corresponds to the mass leaking as gas during the ignition process and is related to the dry weight:

$${\text{Loss }}\;{\text{on}}\;{\text{ ignition }} = \, \left( {{\text{dry }}\;{\text{weight }}{-}{\text{ ashed }}\;{\text{weight}}} \right) \, *{ 1}00 \, /{\text{ dry }}\;{\text{weight}}$$

The three parameters ‘soil moisture’, ‘pH value’ and ‘organic content’ were subjected to statistical analysis. The statistical analysis was conducted using the programme SPSS (IBM SPSS Statistics for Windows, Version 23.0. Armonk, NY).

Due to the high number of zero-values regarding the presence of biting midges, the analyses were based on the bi-weekly sum of *Culicoides* numbers and the means of each soil factor per biotope.

While the soil moisture factor represents a fast changing environmental variable, the pH value and the organic content are known to be relatively constant factors, which usually only vary over a longer time period. Therefore, the only slowly varying pH value and organic content obtained for the three sampling dates 12 June, 23 July and 17 September 2014 were regarded representative for the collection sites throughout the season (interpolation of data).

The three soil factors and the number of *Culicoides* spp. were individually tested for normal distribution utilizing the Kolmogorov–Smirnov-test. For a better understanding of the constitutions of the biotopes, the Kruskal–Wallis-test (as a non-parametric test for not-normally distributed data) was used, and the means of each soil factor for each biotope was calculated. The test was also chosen to check for differences regarding the number of collected *Culicoides* spp. between the four biotopes, followed by a median-test.

The logistic regression model was used to assess the influence of each soil factor on the probability of *Culicoides* spp. to occur. As strong correlations between the soil factors were measured, the regression for each soil factor was calculated separately. A bivariate correlation analysis after Pearson was conducted to examine whether the number of collected *Culicoides* spp. correlated with any of the soil factors.

### Biodiversity indices

To assess the diversity of the studied biotopes and the examined midge fauna, the Shannon–Weaver index (H), the Evenness (E) and the Simpson index (D) were calculated. All biotopes were subject to diversity analysis, except for the “ungrazed meadow”, as no *Culicoides* were detected.

The Shannon–Weaver index was calculated as follows:

$${\text{H }} = \, - \sum {\text{ p}}_{{\text{i}}} *{\text{ log}}_{{2}} {\text{p}}_{{\text{i}}}$$

with p_i_ = the proportion of the total sample represented by species i and p_i_ = n_i_ / N.

For further calculation, the maximum diversity possible (H_max_) was established:

$${\text{H}}_{{{\text{max}}}} = \, - {\text{ log}}_{{2}} {\text{S}}$$

with S = number of detected species (species richness), so that the Evenness (E) could be calculated:

$${\text{E }} = {\text{ H }}/{\text{ H}}_{{{\text{max}}}}$$

The Simpson index, measuring the probability of two individuals randomly selected from one sample to belong to the same species, was calculated as follows:

$${\text{D }} = \, \sum {\text{ n }}\left( {{\text{n}} - {1}} \right) \, /{\text{ N }}\left( {{\text{N}} - {1}} \right)$$

N = number of species.

### Ethics declaration: research involving human and animal participants

This study did not involve protected animal species and no human participants were involved in the work.

### Infomed consent

All authors consent to submission of this manuscript.
